# Letter to the editor regarding: ‘clinical utility of the platelet-to-neutrophil ratio in differentiating sepsis from neonatal pneumonia: an observational study’

**DOI:** 10.1080/07853890.2025.2610904

**Published:** 2025-12-31

**Authors:** Abdullah Akkuş

**Affiliations:** Department of Pediatrics, Necmettin Erbakan University, Konya, Turkey

We read with great interest the recently published article by Li et al. evaluating the clinical usefulness of the platelet-to-neutrophil ratio (PNR) in distinguishing neonatal sepsis among infants with pneumonia [1]. Neonatal sepsis remains one of the leading causes of global infant mortality. Although blood culture is accepted as the diagnostic gold standard, delays in reporting and limited sensitivity create considerable challenges in time‑sensitive neonatal care where early recognition is essential [2].

The authors report that PNR demonstrates fair diagnostic accuracy, with an AUC of 0.76 and moderate sensitivity and specificity. While these findings underscore the feasibility of PNR as an inexpensive and readily available biomarker, the moderate sensitivity suggests limitations as a standalone diagnostic measure in high‑risk neonatal populations. Given that neonates frequently present with subtle or nonspecific symptoms, biomarkers with higher sensitivity are generally preferred to reduce missed diagnoses [3].

The biological rationale supporting PNR is sound. Thrombocytopenia is well recognized in sepsis, reflecting platelet activation, consumption, and sequestration, and is associated with worse outcomes [4]. Likewise, neutrophil dysregulation constitutes a central component of early sepsis pathophysiology. Because PNR integrates these opposing hematologic responses, its use as an adjunctive marker is conceptually justified.

Li et al. further note that PNR outperforms other hematologic ratios previously investigated. However, individual indices capture only select aspects of the immune response. In line with emerging evidence supporting multimarker approaches in sepsis, future research should assess whether combining PNR with additional biomarkers improves diagnostic accuracy [5].

In conclusion, the study contributes meaningful evidence supporting PNR as a promising adjunctive tool for early detection of neonatal sepsis in the setting of pneumonia. Prospective multicenter studies incorporating serial measurements and multivariable biomarker models will further clarify its optimal clinical applicability.

## Data Availability

Data sharing does not apply to this article as no data was created or analyzed in this study.
